# Non-invasive *in vivo* molecular imaging of intra-articularly transplanted immortalized bone marrow stem cells for osteoarthritis treatment

**DOI:** 10.18632/oncotarget.21315

**Published:** 2017-09-27

**Authors:** Bou-Yue Peng, Chi-Sheng Chiou, Navneet Kumar Dubey, Sung-Hsun Yu, Yue-Hua Deng, Feng-Chou Tsai, Han-Sun Chiang, Ying-Hua Shieh, Wei-Hong Chen, Win-Ping Deng

**Affiliations:** ^1^ Oral and Maxillofacial Surgery Section, Department of Dentistry, Taipei Medical University Hospital, Taipei, Taiwan; ^2^ School of Dentistry, College of Oral Medicine, Taipei Medical University, Taipei, Taiwan; ^3^ Division of Allergy, Immunology and Rheumatology, Department of Internal Medicine, Taipei Medical University Hospital, Taipei, Taiwan; ^4^ Stem Cell Research Center, College of Oral Medicine, Taipei Medical University, Taipei, Taiwan; ^5^ Graduate Institute of Biomedical Materials and Tissue Engineering, College of Biomedical Engineering, Taipei Medical University, Taipei, Taiwan; ^6^ Graduate Institute of Medical Sciences, College of Medicine, Taipei Medical University, Taipei, Taiwan; ^7^ Department of Life Science, Fu Jen Catholic University, Taipei, Taiwan; ^8^ Department of Stem Cell Research, Cosmetic Clinic Group, Taipei, Taiwan; ^9^ Graduate Institute of Basic Medicine, Fu Jen Catholic University, Taipei, Taiwan; ^10^ Department of Family Medicine, School of Medicine, College of Medicine, Taipei Medical University, Taipei, Taiwan; ^11^ Department of Family Medicine, Taipei Medical University Hospital, Taipei, Taiwan

**Keywords:** osteoarthritis (OA), immortalized bone marrow stem cells (iBMSCs), cartilage regeneration, human papilloma virus (HPV)-16 E6/E7, molecular imaging

## Abstract

Pathophysiology of osteoarthritis (OA) is characterized by progressive loss of articular cartilage in the knee-joints. To impart regenerative ability in lowly metabolizing chondrocytes, the bone marrow stem cells (BMSCs) has recently been recognized as a superior alternative treatment for OA. However, study of primary BMSCs-mediated chondrogenesis is difficult due to progressive cellular aging and replicative senescence. To obtain a therapeutic cell population for OA, BMSCs were immortalized by human papilloma virus (HPV)-16 E6/E7 along with mCherry luciferase (mCL), a gene marker for non-invasive imaging, and designated as iBMSCs-mCL. Next, their cell morphology, population doubling time (PDT) and colony forming ability (CFU) were evaluated. Furthermore, pluripotency and immunophenotypic markers were investigated. To deduce therapeutic ability, iBMSCs-mCL were intra-articularly injected into right knee of anterior cruciate ligament transaction (ACLT)-OA mice model and tracked through non-invasive bioluminescence imaging. Cell morphology of iBMSCs-mCL was similar to parental BMSCs. PDT and CFU ability of iBMSCs-mCLs were significantly increased. Pluripotency and immunophenotypic markers were highly expressed in iBMSC-mCL. Long-term survival and tri-lineage differentiation particularly chondrogenic potential of iBMSCs-mCL were also demonstrated *in vitro* and then *in vivo* which was monitored through non-invasive imaging. Intensive bioluminescent signals in iBMSCs-mCL administered knee-joint indicated a marked *in vivo* survival and proliferation of iBMSCs-mCL. Immunohistochemical staining for type II collagen (IHC of Col II) and alcian blue & safranin o staining of proteoglycans also corroborated cartilage regeneration by iBMSCs-mCL. Conclusively, iBMSCs-mCL maintains stemness and *in vivo* cartilage regeneration potential suggesting a promising avenue for development of OA therapeutics.

## INTRODUCTION

Osteoarthritis (OA) is characterized by the senescence of articular chondrocytes with aging and deterioration of cartilaginous matrix contributing to structural aberrations [1]. Due to lack of vasculature, the articular cartilage possessing sparse population of chondrocytes is unable to mount an adequate healing response to cartilaginous injury. Moreover, the unique characteristics, complexity and difficult recovery of articular cartilage injury has drawn attention towards advanced alternative therapeutic strategies.

Mesenchymal stem cells (MSCs) has shown the regenerative efficacies in OA treatment due to its multipotency [2]. Bone marrow-derived MSCs (BMSCs) can be induced to chondrogenic lineage under various cultural conditions [3–5]. Previously, we demonstrated stage-specific chondrogenesis of BMSCs through chondrocytic commitment with detailed mechanistic insight [4]. However, BMSCs could lose its phenotype, differentiation potential and finally the termination of proliferation during long-term *in vitro* cultures [6]. The rapid expansion of autologous MSC in a short duration also currently seem impossible [7]. The limited life span of stem cells also represents a hurdle in pre-clinical investigation and therapeutic development. To overcome such limitations, attempts have been made to generate cell lines displaying stable stem cell phenotypes and unlimited proliferation.

For immortalizations, transduced genes such as telomerase reverse transcriptase (TERT) and SV-40LT have been widely utilized. However, disadvantages including cell hypertrophy, senescence, and genetic instability were shown [8, 9]. Previously, we developed an immortalized human articular chondrocytes by employing human papillomavirus (HPV)-16 E6 and E7 genes (designated as hPi cells) for cartilage repair [10], and might be used for differentiating BMSCs to chondrogenic lineage [4]. Additionally, we established an immortalized human nucleus pulposus (ihNP) providing a chondrogenic recovery model for screening regenerative therapeutics [11]. In current research, this promising HPV-16 E6/E7 approach was subsequently utilized to create an immortalized human BMSC to preserve their inherent phenotypes for preclinical study.

To track *in vivo* behavior of transplanted stem cells is an important issue to be addressed. Stem cells labeled with iron oxide nanoparticles can be tracked in arthritic joints for noninvasive diagnosis [12]. However, use of nanoparticles such as superparamagnetic iron oxide (SPIO) showed inhibited chondrogenesis [13] and phenotypical aberrations [14]. We have previously used reporter gene-expressed stem cells or progenitor cells to detect their survival [15–17]. The bioluminescence molecular imaging (BMI) techniques hybridized with luciferase gene are currently being employed to non-invasively trace the *in vivo* cell proliferation and survival over months [18].

This study focuses on establishing immortalized BMSCs with mCherry and luciferase genes (iBMSCs-mCL), to preserve high growth rate, pluripotent marker expression, differentiation potential and prolonged life span. The possible therapeutic effect of iBMSCs-mCL could be demonstrated through its survival, chondrogenic potential and promotion of cartilage regeneration in OA model monitored by *in vivo* imaging system.

## RESULTS

### Characterization of BMSCs after immortalization

**Figure 1:**
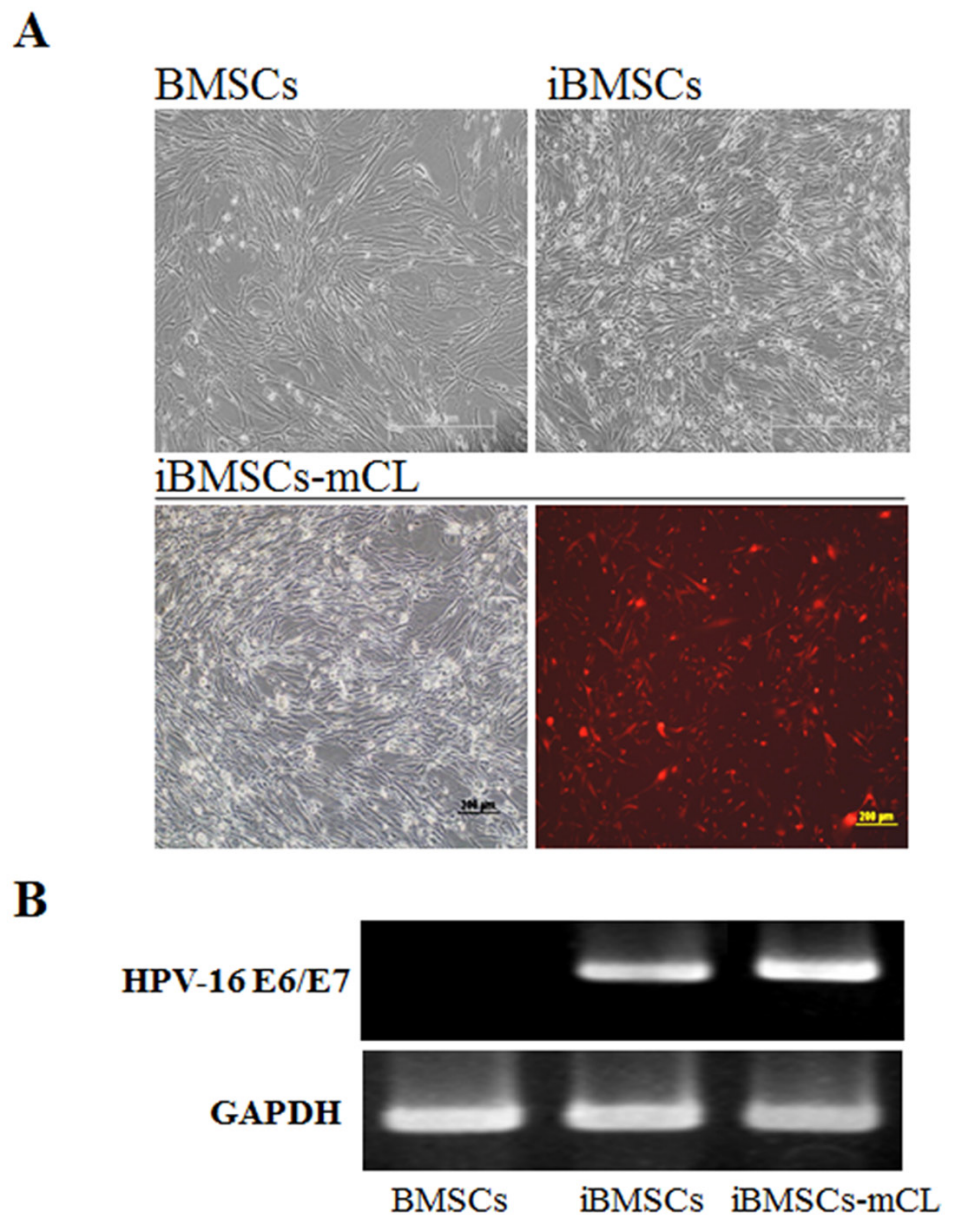
Characterization of immortalized human bone marrow mesenchymal stem cells (iBMSCs) **(A)** Morphology of BMSCs, immortalized BMSCs (iBMSCs) and iBMSCs with luciferase and mCherry (iBMSCs-mCL). Scale bar = 200μm. **(B)** RT-PCR product electrophoresed in 2% agarose gel for the detection of HPV-16 E6/E7. BMSCs were used as a control group while GAPDH as internal standards for RT-PCR.

To establish an immortalized cell line, the amphotropic retroviral vector LXSN16E6E7 was used to transduce the first passage of primary BMSCs. The immortalized BMSCs were designated as iBMSCs and further transduced with imaging selection markers including luciferase and mCherry (iBMSsC-mCL). The iBMSCs and iBMSCs-mCL both displayed a spindle-shaped and fibroblast-like morphology at passage 25 resembling the parental BMSCs at passage 1, and also showed bioluminescence signal (Figure 1A). The results of RT-PCR analysis confirmed the presence of HPV-16 E6/E7 gene in iBMSCs and iBMSCs-mCL with a distinct band at 628 bp after 25 passages while no band was detected in the parental BMSCs (Figure 1B).

### Cell growth and pluripotency of iBMSCs

**Figure 2:**
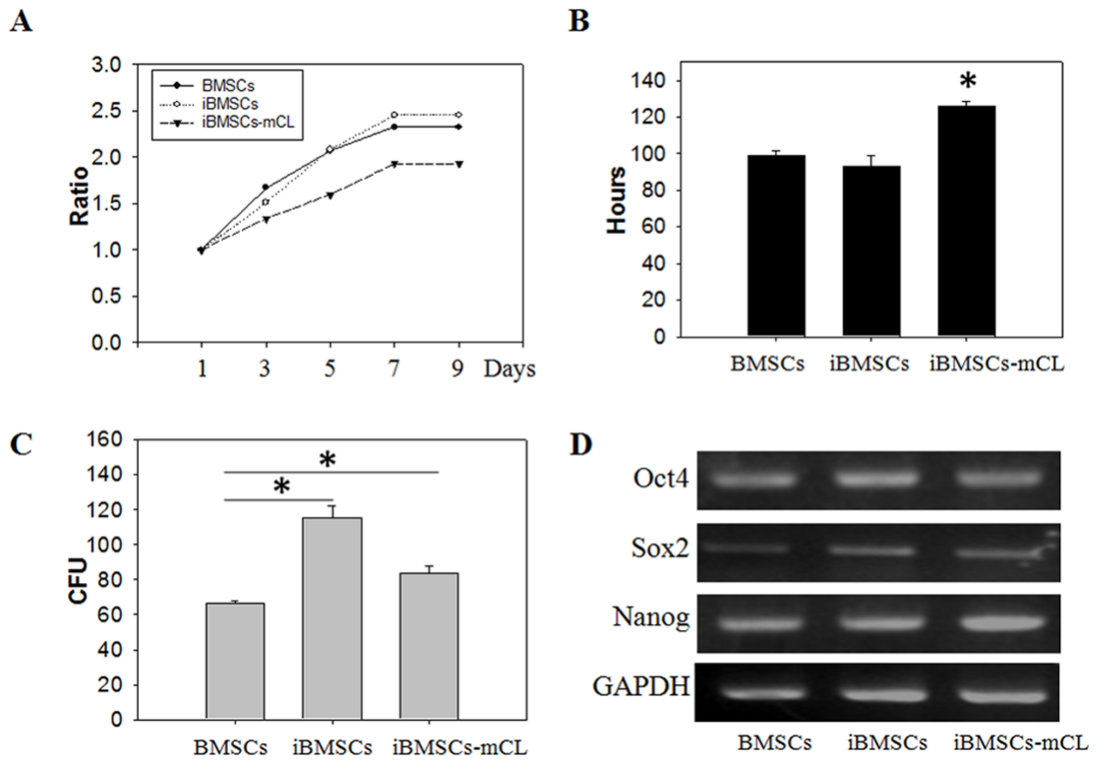
Cell growth and pluripotent potentials in iBMSCs-mCL **(A)** Cell viability, **(B)** population doubling time (in hours), **(C)** colony forming unit (CFU) analysis and **(D)** expression of pluripotency genes including Oct4, Sox2 and Nanog among BMSCs, iBMSCs and iBMSCs-mCL. The GAPDH was used as internal standards for RT-PCR. Results are shown as the mean±SD for three independent experimental cultures using T-test. ^*^p < 0.05.

The cell growth and pluripotent markers of the iBMSCs were then examined. The parental BMSCs and iBMSCs showed a similar proliferation curve, which was higher than that of iBMSCs-mCL (Figure 2A). The PDT of iBMSCs-mCL (126.2±2.4 hr) was longer than the parental BMSCs (98.7±2.9 hr) and iBMSCs (92.9±6.2 hr) (Figure 2B). The CFU in iBMSCs (115.5±6.8) and iBMSCs-mCL (84.0±3.7) were both higher than that of the parental BMSCs (66.7±0.9) (Figure 2C).

The maintenance of pluripotency markers expressed in immortalized BMSCs were determined by RT-PCR (Figure 2D). Compared to parental BMSCs, both the iBMSCs and iBMSCs-mCL expressed slightly higher levels of the Oct4, Sox2 and Nanog, indicating their preserved pluripotency after immortalization.

### Characterizations of cell-surface CD markers on iBMSCs

**Figure 3:**
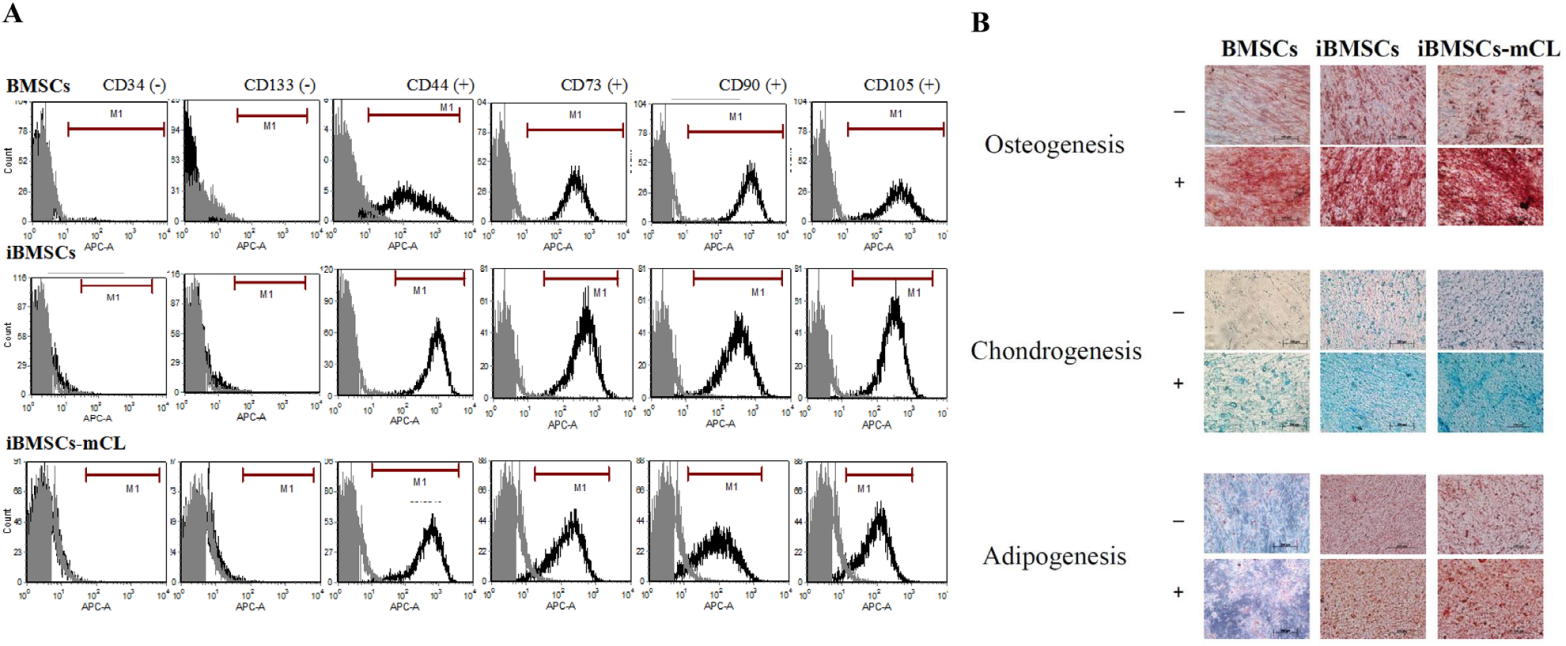
Characterization of BMSCs and its derivatives **(A)** Immunophenotypes were determined by flow cytometry and the respective isotype control is shown as gray. **(B)** Differentiation potentials of immortalized human MSCs. Tri-lineage differentiation potential towards osteogenesis (upper panel), chondrogenesis (middle panel) and adipogenesis (lower panel) among BMSCs, iBMSCs and iBMSCs-mCL treated with (+) or without (-) induction medium. Scale bar: 200μm.

To determine the immunophenotypes, six antibodies against different CD markers were employed in both parental BMSCs and immortalized BMSCs. Flow cytometric analysis revealed that iBMSCs and iBMSCs-mCL both were highly positive for the surface markers CD44, CD73, CD90, CD105 and very low for hematopoietic cell markers including CD34 and CD133, similar to the parental BMSCs (Figure 3A). The results indicated that both the iBMSCs and iBMSCs-mCL express immunophenotypic characteristics of BMSCs.

### Tri-lineage differentiation potential of iBMSC

The cultured parental BMSCs and iBMSCs were tested for their tri-lineage (osteogenic, chondrogenic and adipogenic) differentiation potential under induction conditions and were identified by specific staining. As shown in Figure 3B, in the case of osteogenic differentiation, parental BMSCs, iBMSCs, and iBMSCs-mCL were all demonstrated to have calcified bone matrix stained with brown to dark red by using Alizarin red S. During chondrogenic differentiation, the alcian blue staining revealed the deposition of cartilaginous proteoglycans among all groups. In addition, adipogenic differentiation was evidenced by oil red O which stained the lipid vacuoles in bright red.

### Non-tumorigenicity of iBMSCs in NOD/SCID mice

**Figure 4:**
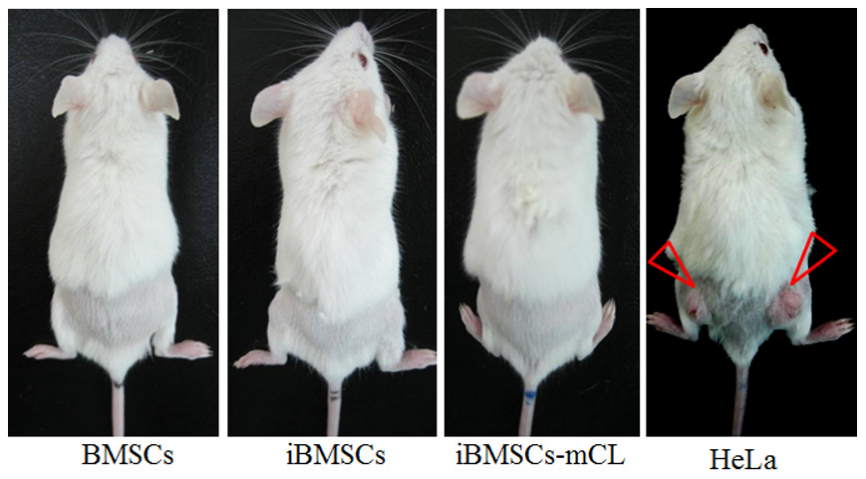
Tumorigenicity assay The mice (NOD/SCID) were subcutaneously injected with BMSCs, iBMSCs, iBMSCs-mCL and HeLa cells respectively. The tumor mass was only observed in mice injected with HeLa cells (indicated by triangular red arrows).

After transduction of HPV-16 E6/E7, the immortalized BMSCs were examined for *in vivo* tumorigenicity. 4×10^6^ cells of parental BMSCs, iBMSCs and iBMSCs-mCL were respectively injected subcutaneously into NOD/SCID mice, while HeLa cells (a malignant cancer cell line) was used as a positive control (Figure 4). No tumor formation was observed among parental BMSCs, iBMSCs and iBMSCs-mCL groups after three-month. After 3-days, tumor masses were only found on dorsa of HeLa-injected mice. Therefore, the iBMSCs and iBMSCs-mCL were both suggested to be immortalized without neoplastic transformation.

### Cartilage regeneration by iBMSCs-mCL for OA monitored by non-invasive bioluminescence imaging (BLI)

**Figure 5:**
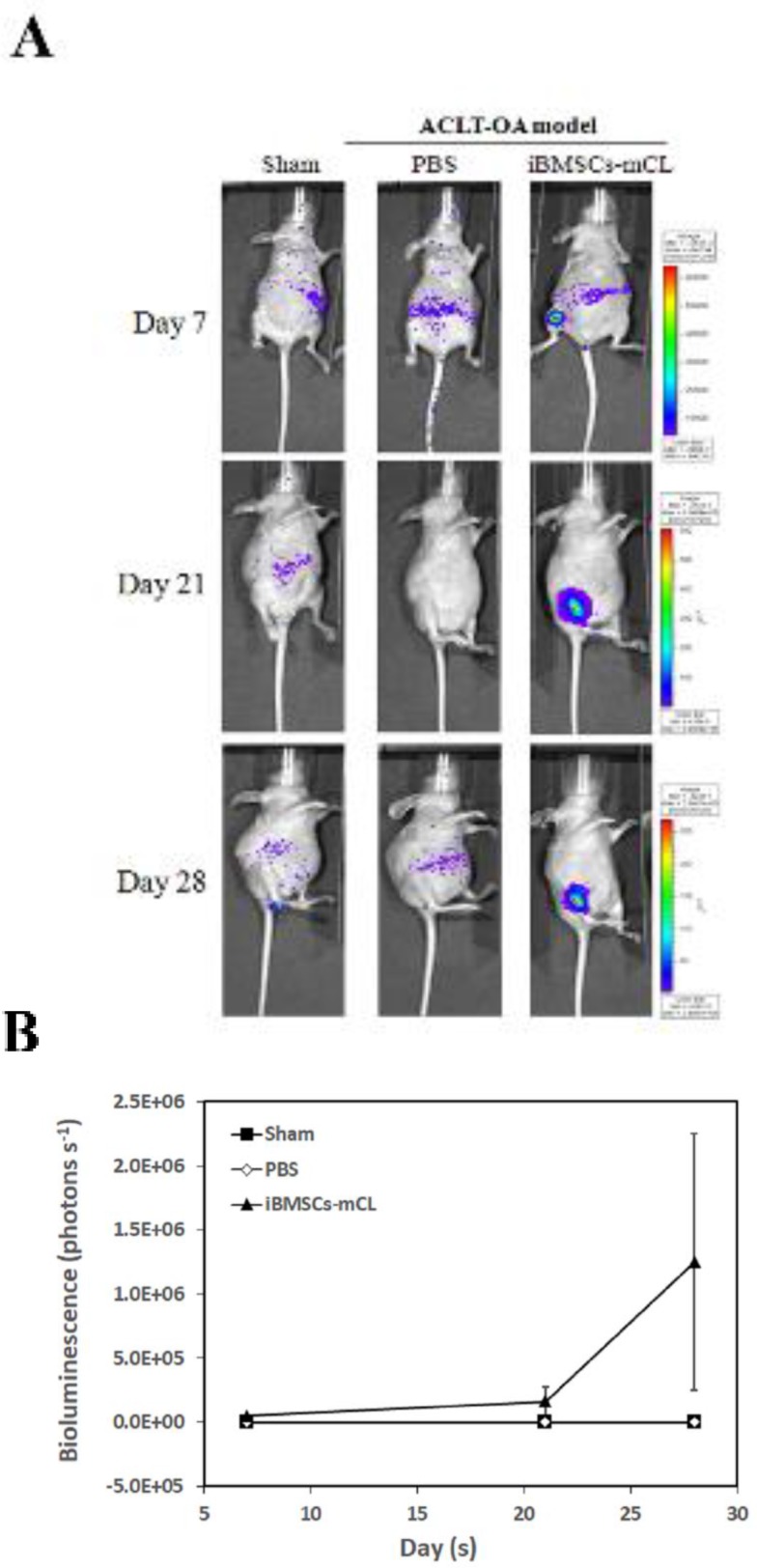
Bioluminescent imaging of anterior cruciate ligament transaction induced-osteoarthritis (ACLT-OA) mice injected with iBMSCs-mCL **(A)** *In vivo* bioluminescence of sham group (left panel), establishment of ACLT-OA for one month and then injected with PBS (middle panel) and iBMSCs-mCL (right panel) for another one month. These mice were then non-invasively detected using IVIS200 for tracing injected cells. **(B)** Quantification of imaging as mean of each group.

Above results showed that iBMSCs-mCL preserved stem cell features and was then utilized for cell-based OA therapy. First, OA mice were created by anterior cruciate ligament transaction (ACLT) in the right knee, as shown in our previous study [19]. The iBMSCs-mCL were injected into right knee of ACLT-OA mice while sham and PBS injection were performed as the control groups (Figure 5A). The *in vivo* BLI was used for tracking and confirming the injected iBMSCs-mCL in the knee of ACLT-OA mice. During day 7 to 28, neither sham nor PBS group revealed specific signals in the right knee. However, the intensive signals were detected and quantified (Figure 5B) in the right knee of iBMSCs-mCL-injected mice, indicating the survival and growth of injected iBMSCs-mCL.

### Histological assessment of cartilage repair by iBMSCs-mCL

**Figure 6:**
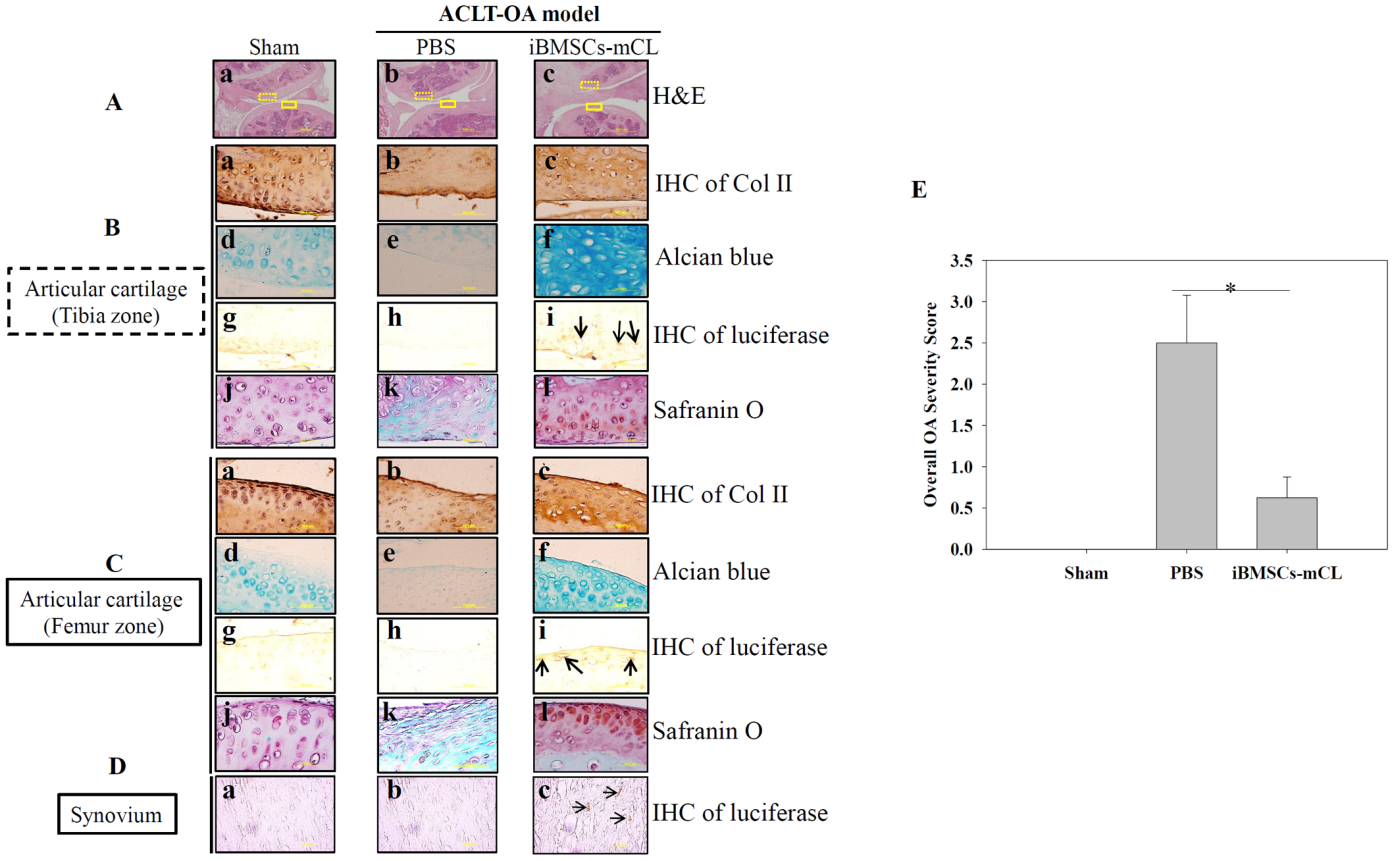
Therapeutic indication of iBMSCs-mCL for OA therapy **(A)** H & E staining of articular cartilage (Aa-c) (100X) in tibia zone (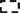) and femur zone (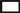) in sham, PBS and iBMSCs-mCL groups. **(B)** and **(C)** Staining of articular cartilage in tibia zone and femur zone respectively. Type II collagen (Col II) (tibia zone: Ba-c; femur zone: Ca-c), Alcian blue staining for proteoglycans (tibia zone: Bd-f; femur zone: Cd-f) and IHC staining of luciferase (tibia zone: Bg-i; femur zone: Cg-i; arrows indicated) (1000X), safranin o staining (tibia zone: Bj-l, femur zone: Cj-l). **(D)** IHC staining of luciferase (synovium: Da-c). **(E)** Comparative histologic scores of OA among sham, PBS and iBMSCs-mCL groups. ^*^ denotes the significant difference as P<0.05.

Since the iBMSCs-mCL could survive in the OA knee for one month, we further examined whether these surviving cells could promote articular cartilage regeneration. The mice were sacrificed and the treated knee joints of mice in each group were harvested. Since direct abrasion occurred in knee joint during OA progression after ACLT surgery, both cartilage surfaces of tibia and femur zone, respectively were then histologically examined (Figure 6). The H&E staining in PBS injected group exhibited a severe degeneration including irregularities in tibia zone and femur zone (H&E panel) with the loss of type II collagen (IHC of Col II) and proteoglycan (alcian blue and safranin o panel). Notably, the iBMSCs-mCL injected group, displayed smooth surfaces of both the tibial and femur cartilage (Figure 6A-c), similar to sham group (Figure 6A-a). Differentiated cells surrounded by lacunae, specific chondrocytic characteristics, were also observed in iBMSCs-mCL group. In addition, intensive signals detected in iBMSCs-mCL group with IHC staining of Col II (Figure 6B-c and 6C-c) and Alcian Blue staining (Figure 6B-f and 6C-f) indicated enhanced cartilaginous matrices than PBS group (Figure 6B-b, 6C-b, 6B-e and 6C-e). Numerous immunopositive luciferase cells were also detected nearby the cartilage surfaces in both tibia (Figure 6B-i), femur zone (Figure 6C-i) and synovium (Figure 6D-c), suggesting the localization of injected iBMSCs-mCL. Furthermore, as demonstrated by safranin o staining, intense red color deposits in articular cartilage matrix of iBMSCs-mCL-injected group (Figure 6B-l and 6C-l) revealed higher accumulation of sulfated proteoglycan compared to PBS (Figure 6B-k and 6C-k) and sham group (Figure 6B-j and C-j). Further, based on safranin o staining, the overall severity scores was found to be higher in PBS group which was reduced in iBMSCs-mCL group (Figure 6E). Collectively, the histologic results indicated that the iBMSCs-mCL possess stronger regenerative efficacy for articular cartilage in an ACLT-OA mice model, supporting the imaging results of cellular growth and survival.

## DISCUSSION

This study aimed to establish an immortalized, stable, multipotent and non-invasively screenable BMSC to attain a cell-based therapeutic alternative to OA. MSCs seem to provide a permanent biological solution due to their proliferative, chondrogenic potential and immunomodulatory nature [20–22]. However, the limited *in vitro* life span of primary MSCs may represent an obstacle for preclinical investigation and future therapeutic development [23]. To overcome these limitations, various oncogenes have been transduced into primary cells for immortalization. Previously, *SV40 large T* (SV40-LT) has been frequently reported with the loss of specific phenotypes in the immortalized cell lines [24]. Additionally, the application of hTERT gene could induce malignant phenotype in prolonged cultures of immortalized cell lines [25]. HPV-16 E6/E7 has also been reported to successfully immortalize lacrimal gland epithelial cells [26] and keratinocytes [27]. The E6 and E7 proteins bind to tumor suppressor transcription factor p53 and retinoblastoma (RB) gene product, respectively. These interactions degenerate the tumor suppressor proteins via ubiquitin-proteasome pathway leading to cell cycle progression [28]. Our previous studies showed that the HPV-16 E6/E7-immortalized chondrocytes cell lines, hPi and ihNP cells could maintain stable chondrogenic phenotypes and stronger regenerative efficacy [10, 11]. These immortalized chondrocyte cell lines may also be used to study stage-specific chondrogenesis and as a screening platform for regenerative therapeutics. The immortalization of stem cell is done to bypass the bottlenecks pertaining to limited life span, maintenance of high cell viability, immunophenotypes and differentiation potential [29, 30]. Thus, we employed an amphotropic retroviral vector, LXSN16E6E7, to transduce HPV-16 E6/E7 genes into primary BMSCs to achieve a successful immortalization. To track the implanted cells, an imaging selection marker, the modified lentiviral vector (FUW-Luc-mCh-puro) [31] was then transduced into the immortalized BMSCs, designated as iBMSCs-mCL.

Over 25 passages, the iBMSCs-mCL still displayed the fibroblastic morphology and preserved the self-renewal potential, similar to parental BMSCs (Figures 1A and 2). The population doubling time of iBMSCs-mCL was slightly longer than the parental BMSCs and iBMSCs. Furthermore, in previous studies, decreased expression levels of the pluripotent transcription factors and cell differentiation markers have been reported [32]. However, the pluripotency-specific transcription factors including Oct4, Sox2 and Nanog (Figure 2D) were still preserved, verifying their persistent pluripotent nature of iBMSCs-mCL. These factors are responsible for maintenance of pluripotency and self-renewal of stem cells phenotypes, as well as characteristics of parental BMSCs [33–36]. To identify stem cells, the MSCs population display a positive expression profile of specific surface antigens, including CD44, CD73 (5’–ectonucleotidase), CD90 (Thy-1), CD105 (endoglin) and negative expression of hematopoietic stem cell immunophenotypes including CD34, CD45 and CD133 [37, 38]. During *in vitro* manipulation, primary stem cells may also change their immunophenotypic markers which alter their inherent properties [39, 40]. We have established an iBMSC-mCL without changing immunophenotype of MSCs profiles as demonstrated by high expressions of CD44, CD73, CD90 and CD105 and feeble expressions of CD34 and CD133 (Figure 3A), all of which remained unaltered over the 25 passages.

Another critical characteristic of stem cells is to differentiate into osteoblasts, chondrocytes and adipocytes *in vitro* [37]. Differentiation plasticity might be lost in BMSCs after immortalization [41]. However, our study showed that iBMSCs and iBMSCs-mCL could maintain multi-differentiation abilities including osteogenic, chondrogenic and adipogenic lineages, as demonstrated by biochemical stains (Figure 3B). This result is in agreement with the study by Cecilia et al., in which using HPV-16 E6/E7 genes, an immortalized, stable and multi-potent epithelial stem cell line have already been established [42]. Additionally, transformation of immortalized cell to tumorigenic phenotype might also be associated [43]. However, the iBMSCs-mCL showed no tumor formation *in vivo* (Figure 4). Collectively, these results support the preclinical application of iBMSCs-mCL for tissue regeneration.

Since many immortalized human BMSCs have been successfully established, their applications are still unclear yet [44, 45]. Hence, the *in vivo* efficacy of iBMSC-mCL was further examined. We have previously investigated the regenerative efficacies of stem cells for cartilage engineering [4, 10]. We created *in vivo* OA model through ACLT surgical procedure [19], and after confirming the native characteristics of iBMSCs-mCL, the modified lentiviral vector with dual imaging genes (FUW-Luc-mCh-puro) was then transduced into iBMSCs and administered for *in vivo* cartilage regeneration in the ACLT-OA mice model. Notably, monitoring of *in vivo* behavior and survival of implanted MSCs in host tissue is essential for development of successful cell therapy [46]. In our previous study, the bioluminescence imaging by IVIS technology has already been successfully established to non-invasively monitor the transplanted cells [47]. We demonstrated the growth and survival of iBMSCs-mCL in the ACLT-OA mice by examining enhanced intensive signals through IVIS imaging after 1-month implantation (Figure 5), indicating their continued proliferation under *in vivo* conditions. This results is in the agreement with study by Jonathan et al. in which E6 have been reported to bypass replicative senescence to stabilize and elongate the telomeres leading to enhanced proliferative capacity [48]. The therapeutic efficacy of localized iBMSCs-mCL in OA knee-joint was demonstrated through an intact and smoother cartilage surfaces along with higher proteoglycans in the iBMSCs-mCL-injected group (Figure 6). We have previously demonstrated the commitment of BMSCs towards stage-specific chondrogenesis [4]. Accordingly, iBMSCs-mCL group showed intensive signals in accumulated matrix of regenerated tissues, indicating the abilities of improved tissue filling and accelerated matrix synthesis. Since iBMSCs-mCL could show regenerative potential, the cell line might also provide trophic factors for resident chondrocytes in restoring architecture of articular cartilage. Interestingly, Balducci et al. demonstrated a similar phenotypic profile and enhanced ability to secrete high levels of angiogenic factors by E6/E7 immortalized mesenchymal adipose-derived stromal cells [23]. Moreover, another report by Chang et al. showed that E6/E7 mediated immortalized MSC released IL-1β and VEGF-A which are important paracrine factors to enhance the angiogenesis via AKT activation thereby ameliorating limb ischemia [49]. These previous reports are an indicative of a possible positive effect of E6/E6 based immortalization on secretion of immunomodulatory/trophic factors. However, the additional studies are required to determine the relative direct versus indirect contributions of the iBMSCs in preventing OA progression after joint injury.

Conclusively, we elucidated that iBMSCs-mCL could survive in OA knee more than 1 month with stronger regenerative potential after intra-articularly transplanted in OA animal model. Further, the iBMSC-mCL could differentiate into chondrocyte-like cells and synthesize ECM to repair OA knee cartilage. Finally, we demonstrated that in addition to the fundamental features of primary BMSCs, the iBMSCs-mCL possesses higher viability and multi-differentiation potential, and could be a promising candidate for cartilage regeneration to provide a therapeutic solution to OA.

## MATERIALS AND METHODS

### Culture and maintenance of BMSCs

Human BMSCs were kindly provided by Dr. Shiaw-Min Hwang. For routine cultures, BMSCs were maintained in α-MEM supplemented with 20% fetal bovine serum (FBS; Hyclone, Logan, UT) in a humidified atmosphere containing 5% CO_2_. The cells were seeded at a density of 6×10^3^ cells/cm^2^, passaged twice a week, and the culture medium was changed every two days. Finally, the BMSCs (passage 3̴ 5) were selected for immortalization to be used in further experiments.

### Retroviral vector transduction with HPV-16 E6/E7 gene and integration of modified lentiviral vector with dual imaging selection markers (FUW-Luc-mCh-puro)

The transduction of retroviral vector has been previously described [10]. The infected iBMSCs (designated as iBMSCs-mCL) were then harvested, grown and expanded for their characterization and subsequent functional studies.

### Cell viability

Cell viability was measured using MTT assay (Sigma, USA). Cells were seeded into 96-well plate at a density of 4x10^3^ cells/ well and cultured for 7 days. At day 1, 3, 5, and 7, 20 μL MTT was added to each well and incubated for 4 hours. The medium was then removed and 150 μL DMSO (Sigma, USA) was used to lyse cells. The absorbance of the cell lysates was measured at 570 nm by using a Multiskan PC (Thermo Labsystem). Cell population doubling time (PDT) was then calculated using the following function:

$$\mathrm{PDT}=\left(T-T_{0}\right)\log2/\left(\log N-\log N_{0}\right)$$

T –T_0_ indicates the length of time between two measurements and N_0_ and N denote the OD value at two points of measurement. The experiments performed in triplicate were evaluated.

### Reverse transcription-polymerase chain reaction (RT-PCR)

Total RNA from subconfluent monolayer cultures was extracted using TRIzol^®^ reagent (Invitrogen Life Technologies, Carlsbad, CA, USA) and subjected to RT followed by PCR amplification for specifically expressed genes. RT was performed with SuperScript^™^ III (Invitrogen Life Technologies, Carlsbad, CA, USA) and an Oligo d(T)_12-18_ primer, as previously described [50]. The primers used were:

HPV-16 E6/E7-F: 5’- ATC CAT AGT ATA TAG AGA TGG GAA T- 3’

HPV-16 E6/E7-R: 5’- CTG CAG GAT CAG CCA TGG TAG A- 3’

Human SOX2-F: 5’ -TACAGCATGTCCTACTCGCAG - 3’

Human SOX2-R: 5’ -GAGGAAGAGGTAACCACAGGG – 3’

Human Nanog-F: 5’-CCT CTT AAA TTT TTT CCT CCT CTT C-3’

Human Nanog R: 5’-AAG TGG GTT GTT TGC CTT TG-3’

Human Oct4-F: 5’-CGT GAA GCT GGA GAA GGA GAA GCT G-3’

Human Oct4-R: 5’-CAA GGG CCG CAG CTT ACA CAT GTT C-3’

### Immunophenotyping of iBMSC-mCL

To test immunophenotyping of iBMSC-mCL, cells were trypsinized, washed, and resuspended in PBS at a density of 10^6^ cells/ ml. After fixation, cells were washed twice and cell pellets were resuspended in 0.5ml PBS containing primary antibody for 30 minutes. Cells were immunolabeled with the following mouse anti-human antibodies: CD34, CD44, CD73, CD90, CD105 (eBioscience, San Diego, CA), and CD133 (MACS, Bergisch Gladbach, Germany). The non-specific mouse IgG (eBioscience, San Diego, CA) was substituted for the primary antibodies as isotype control. Subsequently, cells were washed twice and resuspended in 0.5ml for FACS can flow cytometry (Becton, Dickson and company, San Jose, CA).

### Multilineage potentials

For determining the multilineage potential, iBMSC-mCL were cultured until reaching 90% confluence and then treated with the following induction medium: (a) Osteogenesis: α-MEM supplemented with 10% FBS, 0.1 μM dexamethasone (Sigma), 5 mM β-glycerophosphate (Sigma), and 50 μM ascorbic acid (Sigma); (b) Chondrogenesis: α-MEM supplemented with 10% FBS and 10 ng/ml TGF-β1 (PeproTech, Rocky Hill, NJ); (c) Adipogenesis: α-MEM supplemented with 10% FBS, 1M dexamethasone (Sigma), 0.5 mM isobutyl-methylxanthine (Sigma), 10 μg/ml insulin (Gibco BRL, Carlsbad, CA), and 100 μg/ml indomethacin (Sigma).

After induction process, cells were assayed by specific-matrix staining in which alizarin Red S, alcian blue and oil-red O were used to determine osteo-, chondro- and adipogenic ability respectively.

### Tumorigenicity assay

The animal experiment was conducted in compliance with the protocol approved by the Institutional Animal Care and Use Committee of Taipei Medical University. The iBMSC-mCL were trypsinized and re-suspended at 4×10^6^ cells/mL in PBS and thereafter subcutaneously injected into the dorsa of each NOD/SCID mice (n=6) (obtained from National Taiwan University Laboratory Animal Center, Taipei, Taiwan). Mice were housed in sterilized pathogen-free cages and observed daily for tumor formation over 3 months. HeLa cells and parental BMSCs were injected as positive and negative controls, respectively.

### Creation of transaction of anterior cruciate ligament (ACLT)-induced OA animal model, bioluminescence imaging (BLI), and histopathologic examinations

ACLT-induced OA animal model has already been well-established in our previous study [19]. The experimental OA was induced in 8-week-old female BALB/c nude mice by ACLT in the right knee. 1×10^6^ iBMSC-mCL were intra-articularly injected into the right-knee (n=6) while sham operation or PBS injection (n=6) was used as the control group after one month-ACLT surgery. After another one month, BLI was performed with an IVIS Imaging System 200 Series (PerkinElmer). Firstly, anesthetized mice were injected intraperitoneally with 75 mg/kg of D-Luciferin and images were acquired 2–5 min post-injection. Initial acquisition time was 2 min and later reduced in accordance with signal intensity to avoid saturation.

The images were then quantified with Living Image software (Xenogen) by measuring bioluminescence (photons s^-1^) in regions of interest drawn around appropriate signals.

Finally, animals were sacrificed and treated knee-joints were harvested, fixed in 4% phosphate-buffered formaldehyde, then embedded in paraffin and sectioned for histological examination. Tissue sections were first counterstained with hematoxylin and eosin (H&E) for evaluating the morphological changes. For chondrogenic markers, sections were stained with IHC of type II collagen (Col II, Chemicon International, Temecula, CA, USA) and alcian blue (Sigma) and safranin o (Sigma) stain for proteoglycan content. Luciferase expression was detected by staining with luciferase monoclonal antibody (Chemicon International, Temecula, CA, USA). IHC stains were then reacted with avidin and biotin and peroxidase substrate kits were used to locate immunoreactivity with DAB chromogen (Vector Laboratories). Further, a histologic grading was performed using safranin o staining to evaluate OA status, as described previously [51].

### Statistical analysis

All the results are shown as the mean±standard deviation (SD) for each group (n=6). In the PDT and CFU results, iBMSCs-mCL was compared with BMSCs or iBMSCs using student *t-test*. From the *in vivo* model, iBMSCs-mCL group was compared with sham or PBS groups using student *t-test*. A *p-value*<0.05 was considered as a significant difference.

## Abbreviations

OA: osteoarthritis
iBMSCs: immortalized bone marrow stem cells
HPV: human papilloma virus-16 E6/E7
PDT: population doubling time
CFU: colony forming ability
ACLT: anterior cruciate ligament transaction
Col II: type II collagen
IHC: immunohistochemistry
BLI: bioluminescence imaging
