# Correction: Non-invasive *in vivo* molecular imaging of intra-articularly transplanted immortalized bone marrow stem cells for osteoarthritis treatment

**DOI:** 10.18632/oncotarget.25501

**Published:** 2018-05-18

**Authors:** Bou-Yue Peng, Chi-Sheng Chiou, Navneet Kumar Dubey, Sung-Hsun Yu, Yue-Hua Deng, Feng-Chou Tsai, Han-Sun Chiang, Ying-Hua Shieh, Wei-Hong Chen, Win-Ping Deng

**Affiliations:** ^1^ Oral and Maxillofacial Surgery Section, Department of Dentistry, Taipei Medical University Hospital, Taipei, Taiwan; ^2^ School of Dentistry, College of Oral Medicine, Taipei Medical University, Taipei, Taiwan; ^3^ Division of Allergy, Immunology and Rheumatology, Department of Internal Medicine, Taipei Medical University Hospital, Taipei, Taiwan; ^4^ Stem Cell Research Center, College of Oral Medicine, Taipei Medical University, Taipei, Taiwan; ^5^ Graduate Institute of Biomedical Materials and Tissue Engineering, College of Biomedical Engineering, Taipei Medical University, Taipei, Taiwan; ^6^ Graduate Institute of Medical Sciences, College of Medicine, Taipei Medical University, Taipei, Taiwan; ^7^ Department of Life Science, Fu Jen Catholic University, Taipei, Taiwan; ^8^ Department of Stem Cell Research, Cosmetic Clinic Group, Taipei, Taiwan; ^9^ Graduate Institute of Basic Medicine, Fu Jen Catholic University, Taipei, Taiwan; ^10^ Department of Family Medicine, School of Medicine, College of Medicine, Taipei Medical University, Taipei, Taiwan; ^11^ Department of Family Medicine, Taipei Medical University Hospital, Taipei, Taiwan

**This article has been corrected:** The 2nd affiliation is added to the author given below:

**Bou-Yue Peng^1,2^**

Original article: Oncotarget. 2017; 8:97153-97164. https://doi.org/10.18632/oncotarget.21315

